# A Drug Combination Screen Identifies Drugs Active against Amoxicillin-Induced Round Bodies of *In Vitro Borrelia burgdorferi* Persisters from an FDA Drug Library

**DOI:** 10.3389/fmicb.2016.00743

**Published:** 2016-05-23

**Authors:** Jie Feng, Wanliang Shi, Shuo Zhang, David Sullivan, Paul G. Auwaerter, Ying Zhang

**Affiliations:** ^1^Department of Molecular Microbiology and Immunology, Johns Hopkins Bloomberg School of Public Health, Johns Hopkins University, BaltimoreMD, USA; ^2^Fisher Center for Environmental Infectious Diseases, School of Medicine, Johns Hopkins University, BaltimoreMD, USA

**Keywords:** *Borrelia burgdorferi*, round bodies, persisters, drug combination drug screen, FDA drug library

## Abstract

Although currently recommended antibiotics for Lyme disease such as doxycycline or amoxicillin cure the majority of the patients, about 10–20% of patients treated for Lyme disease may experience lingering symptoms including fatigue, pain, or joint and muscle aches. Under experimental stress conditions such as starvation or antibiotic exposure, *Borrelia burgdorferi* can develop round body forms, which are a type of persister bacteria that appear resistant *in vitro* to customary first-line antibiotics for Lyme disease. To identify more effective drugs with activity against the round body form of *B. burgdorferi*, we established a round body persister model induced by exposure to amoxicillin (50 μg/ml) and then screened the Food and Drug Administration drug library consisting of 1581 drug compounds and also 22 drug combinations using the SYBR Green I/propidium iodide viability assay. We identified 23 drug candidates that have higher activity against the round bodies of *B. burgdorferi* than either amoxicillin or doxycycline. Eleven individual drugs scored better than metronidazole and tinidazole which have been previously described to be active against round bodies. In this amoxicillin-induced round body model, some drug candidates such as daptomycin and clofazimine also displayed enhanced activity which was similar to a previous screen against stationary phase *B. burgdorferi* persisters not exposure to amoxicillin. Additional candidate drugs active against round bodies identified include artemisinin, ciprofloxacin, nifuroxime, fosfomycin, chlortetracycline, sulfacetamide, sulfamethoxypyridazine and sulfathiozole. Two triple drug combinations had the highest activity against amoxicillin-induced round bodies and stationary phase *B. burgdorferi* persisters: artemisinin/cefoperazone/doxycycline and sulfachlorpyridazine/daptomycin/doxycycline. These findings confirm and extend previous findings that certain drug combinations have superior activity against *B. burgdorferi* persisters *in vitro*, even when pre-treated with amoxicillin. These findings may have implications for improved treatment of Lyme disease.

## Introduction

Lyme disease may affect multiple organs in disseminated infection and is the most common vector-borne infection reported in the United States and Europe. In the United States, the number of reported Lyme disease cases has increased over last 15 years and a recent calculation estimated that about 300,000 cases occur annually (CDC, 2015a). About 10–20% of patients treated for Lyme disease with a recommended 2–4 week antibiotic therapy have lingering symptoms of fatigue, pain, or joint and muscle aches (CDC, 2015a). In some cases, when such symptoms extend beyond 6 months after initial antibiotic treatment, post-treatment Lyme disease syndrome (PTLDS) has been proposed (CDC, 2015b) that in placebo-controlled, randomized trials that does not appear to respond to additional treatment with current Lyme antibiotics (Wormser et al., 2006; Klempner et al., 2013; Berende et al., 2016), though improved symptoms were noted in other trials (Krupp et al., 2003; Fallon et al., 2008).

The spirochete *Borrelia burgdorferi* sensu lato is the causative agent of Lyme disease (Meek et al., 1996). *B. burgdorferi* is transmitted by tick vectors that also feed upon host reservoirs including rodents, reptiles, birds and deer (Radolf et al., 2012). In bacterial culture systems, the morphology of *B. burgdorferi* is mainly in spirochetal form during log phase growth, but transforms to morphological variant forms such as round bodies and microcolonies in older, stationary phase cultures (Feng et al., 2014a, 2015) or when the bacteria are subjected to adverse or stress conditions (Brorson and Brorson, 1997; Murgia and Cinco, 2004). For example, oxidative stress, pH variation, heat and antibiotic exposure can induce *B. burgdorferi* to form round bodies (sometimes referred to as cystic forms which are coccoid and membrane-bound cells) (Kersten et al., 1995; Murgia and Cinco, 2004; Brorson et al., 2009).

*In vitro* studies have shown that the round body form of *B. burgdorferi* is not only viable but also could revert to spirochetal form under suitable conditions (Brorson and Brorson, 1997; Murgia and Cinco, 2004). These round bodies appear to have both lower metabolism and greater resistance to antibiotic treatment, which appears common to stationary phase bacteria in culture generally. Some have suggested that these round body forms might be a protective mechanism to overcome adverse environmental conditions (Murgia and Cinco, 2004; Brorson et al., 2009). Although the round body forms of *B. burgdorferi* have been described in human infections including brain tissue (Miklossy et al., 2008), this finding has not been widely observed in the scientific literature, and their significance in Lyme disease is unclear (Lantos et al., 2014).

Amoxicillin and doxycycline are among the most commonly used frontline drugs for the treatment of Lyme disease, but have poor activity against *in vitro*, stationary phase cultures enriched with persister forms including round bodies and microcolonies (Kersten et al., 1995; Brorson et al., 2009; Barthold et al., 2010; Sapi et al., 2011; Feng et al., 2015). Commonly used Lyme antibiotics doxycycline and penicillin have been shown experimentally to induce *B. burgdorferi* from spirochetal to round bodies or cystic round structures forms in culture (Kersten et al., 1995). In this study, we took advantage of this observation and established a round body persister model using amoxicillin-treated organisms to screen a FDA-approved drug library with a newly developed SYBR Green I/propidium iodide (PI) assay (Feng et al., 2014a,b). We sought to investigate whether some non-traditional drugs or drug combinations had improved activity against the amoxicillin-induced round body form as distinct from the previous drug candidates identified from non-antibiotic treated stationary phase culture (Feng et al., 2014a).

## Materials and Methods

### Strain, Media and Culture

*Borrelia burgdorferi* strain B31 was obtained from American Type Tissue Collection. *B. burgdorferi* and was cultured in BSK-H media (HiMedia Laboratories Pvt. Ltd.), with 6% rabbit serum (Sigma–Aldrich, Co). All culture media were filter-sterilized by 0.2 μm filter. Cultures were incubated in sterile 50 mL closed conical tubes (BD Biosciences, San Jose, CA, USA) at 33°C without antibiotics.

### Induction of Round Body Form of *B. burgdorferi*

For induction of *B. burgdorferi* to round body forms, *B. burgdorferi* spirochetes (1 × 10^5^ spirochetes/ml) were cultured in BKS-H medium for 5 days without shaking when the bacteria are still in log phase (Feng et al., 2014a). After 5 days of incubation, amoxicillin at a final concentration of 50 μg/ml was added to the culture for round body form induction. After 72 h at 33°C, *B. burgdorferi* were examined by microscopy following SYBR Green I/PI staining to confirm round body formation. The round body cells (100 μl) were transferred to 96-well tissue culture microplates for evaluation of the effects of treatment with antibiotics or FDA drug library (see below). To confirm the viability of induced round body form, we subcultured the induced round bodies in fresh BSK-H medium. The round bodies (in 500 μl culture) were collected by centrifugation and rinsed with 1 ml fresh BSK-H medium followed by resuspension in 500 μl fresh BSK-H medium. Then 50 μl of cell suspension was transferred to 1 ml fresh BSK-H medium for subculture at 33°C for 5 days.

### Microscopy Techniques

Specimens were examined on a Nikon Eclipse E800 microscope equipped with differential interference contrast (DIC) and epi-fluorescence illumination, and recorded with a Spot slider color camera. Cell proliferation assays were performed by direct counting using a bacterial counting chamber (Hausser Scientific Partnership, Horsham, PA, USA) and DIC microscopy. To assay the viability of *B. burgdorferi*, the SYBR Green I/PI assay (Feng et al., 2014a) was performed. The ratio of live (green) and dead (red) *B. burgdorferi* was calculated by counting the cells using a bacterial counting chamber and epi-fluorescence microscopy.

### Antibiotics and FDA Drug Library

Doxycycline, metronidazole, cefmetazole, rolitetracycline, sulfachlorpyridazine, artemisinin, cefoperazone, daptomycin (Sigma–Aldrich) were dissolved in suitable solvents (Wikler and Ferraro, 2008) to form stock solutions. The antibiotic stocks were filter-sterilized by 0.2 μm filter, except metronidazole and artemisinin which were dissolved in dimethylsulfoxide (DMSO) and not filtered. Then the stocks were diluted into 500 μM pre-diluted stocks and stored at -20°C.

Each drug in the JHCCL FDA-approved drug library version 1.32 (Chong et al., 2006) was predispensed in 10 mM stock solutions with DMSO. The stock solutions were arrayed in a total of 27 96-well plates, leaving the first and the last columns in each plate as controls. Each solution in these master plates was diluted with PBS to make 500 μM pre-diluted working stock plates. The first and the last columns in each pre-diluted plate were set as blank controls, doxycycline control, metronidazole control and amoxicillin control. The pre-diluted drug stock plates were sealed and stored at -20°C.

### Drug Screen and Antibiotic Susceptibility Testing

To qualitatively determine the effect of antibiotics in a high-throughput manner, 10 μl of each compound (final concentration 50 μM) from the pre-diluted plate or pre-diluted stock was added to 3 days amoxicillin induced round body form from a 5 day-old stationary phase *B. burgdorferi* culture in the 96-well plate. The final volume per well was adjusted to 100 μl. Plates were sealed and placed in 33°C incubator for 7 days. SYBR Green I/PI viability assay was used to assess the live and dead cells after antibiotic exposure as described (Feng et al., 2014a). Briefly, 10 μl of SYBR Green I (10,000 × stock, Invitrogen) was mixed with 30 μl propidium iodide (PI, 20 mM, Sigma) into 1.0 ml of sterile dH_2_O. Then 10 μl staining mixture was added to each well and mixed thoroughly. The plates were incubated at room temperature in the dark for 15 min followed by plate reading at excitation wavelength at 485 nm and the fluorescence intensity at 535 nm (green emission) and 635 nm (red emission) in microplate reader (HTS 7000 plus Bio Assay Reader, PerkinElmer Inc., USA). With least-square fitting analysis, the regression equation and regression curve of the relationship between percentage of live and dead bacteria as shown in green/red fluorescence ratios was obtained. The regression equation was used to calculate the percentage of live cells in each well of the 96-well plate.

The standard microdilution method (Chong et al., 2006) was used to determine the minimum inhibitory concentration (MIC) that would inhibit visible growth of *B. burgdorferi* after 72 h incubation. *B. burgdorferi* cells (1 × 10^5^) were inoculated into each well of a 96-well microplate containing 90 μL fresh BSK-H medium per well. Each diluted compound (10 μL) was added to the culture. All experiments were run in triplicate. The 96-well plate was sealed and placed in an incubator at 33°C for 5 days. Cell proliferation was assessed using the SYBR Green I/PI assay and a bacterial counting chamber after the incubation.

### Subculture of Antibiotic-Treated *B. burgdorferi* to Assess Viability of the Organisms

Amoxicillin-induced round body form and 10-day old stationary phase culture (500 μl) of *B. burgdorferi* was treated with 10 μg/ml drugs or their combinations for 7 days in 1.5 ml Eppendorf tubes as described previously (Feng et al., 2015). After incubation at 33°C for 7 days without shaking, the cells were collected by centrifugation and rinsed with 1 ml fresh BSK-H medium followed by resuspension in 500 μl fresh BSK-H medium without antibiotics. Then 50 μl of cell suspension was transferred to 1 ml fresh BSK-H medium for subculture at 33°C for 10–20 days. Cell proliferation was assessed using SYBR Green I/PI assay and bacterial counting chamber (Hausser Scientific Partnership, Horsham, PA, USA) by microscopy as described above.

## Results

### Induction of Round Body Form of *B. burgdorferi* by Amoxicillin

**FIGURE 1:**
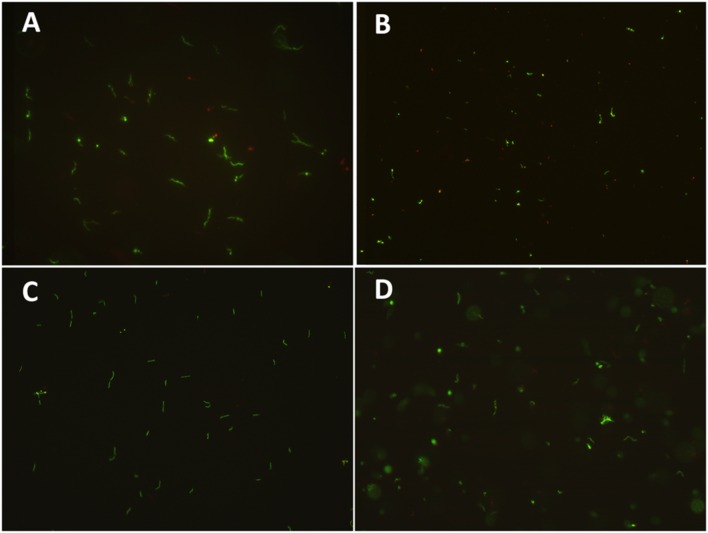
**Microscopy demonstrating round body formation in the presence of amoxicillin and subsequent reversion to spirochetal form of *B. burgdorferi* during subculture.** **(A)** A 5-day old *B. burgdorferi* culture without amoxicillin consisting primarily of spirochetal form. **(B)** Coccoid round body forms formed from *B. burgdorferi* spirochetes upon treatment with amoxicillin (50 μg/ml) for 3 days. **(C)** Reversion of round body form of *B. burgdorferi* from **(B)** to spirochetal form after 5 days subculture in fresh BSK-medium. **(D)** 7-day old stationary phase *B. burgdorferi* treated with 100 μg/ml amoxicillin for 3 days. The images were derived from the above samples stained by SYBR Green PI viability assay followed by microscopy (400 × magnification) as described in Section “Materials and Methods.”

Beta-lactam antibiotics are among the most commonly used drugs for the treatment of Lyme disease, but intriguingly *in vitro* induce spirochetal *B. burgdorferi* to form round bodies that are subsequently resistant to many antibiotics (Brorson et al., 2009; Sapi et al., 2011). In order to identify FDA-library drugs active against the round body form of *B. burgdorferi*, we first assessed conditions that yielded the highest induction of round body forms. The 6-day or older culture could not be induced to round body form completely (<80%) with even 100 μg/ml amoxicillin (**Figure 1D**), whereas a 5-day *B. burgdorferi* culture treated with 50 μg/ml amoxicillin for 72 h was the best condition for producing round bodies. Microscopic examination showed that in 5-day old culture after 3-day amoxicillin induction, up to 96% of the *B. burgdorferi* spirochetes could be induced into round bodies by amoxicillin (**Figure 1A**). To confirm that the induced round body form was still viable, we performed a subculture test in fresh BSK-H medium. Microscopy analysis revealed that amoxicillin-induced round bodies of *B. burgdorferi* could revert to spirochetes (95 ∼ 99%, *n* = 5) in BSK-H medium after 5 days subculture (**Figure 1**), indicating that the round bodies induced by amoxicillin treatment remained viable upon subculture.

**FIGURE 2:**
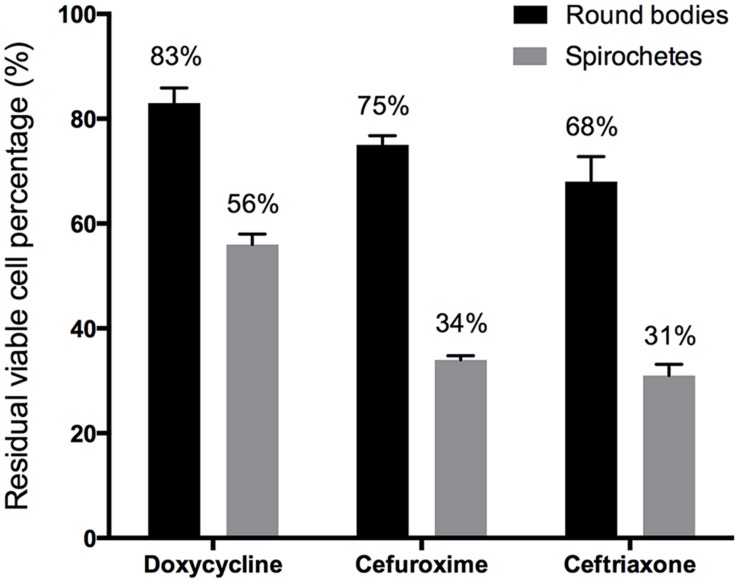
**Exposure of spirochetal form and amoxicillin-induced round bodies of *B. burgdorferi* (5-day old) to different antibiotics.** Log phase spirochetal form (5 day old) and amoxicillin-induced round bodies of *B. burgdorferi* (5-day old) were exposed to 50 μM doxycycline, cefuroxime, and ceftriaxone, respectively, for 5 days. The percentage of residual live cells (*n* = 3) was determined by SYBR Green I/PI assay followed by fluorescence microscopy counting.

To compare the antibiotic susceptibility of the round body form of *B. burgdorferi* with the spirochetal form, we tested commonly used antibiotics for Lyme disease including doxycycline, cefuroxime, and ceftriaxone on 5-day old spirochetes and amoxicillin-induced round body form of *B. burgdorferi*. The results showed that the round body form of *B. burgdorferi* was more tolerant or resistant to antibiotics than the spirochetal form (**Figure 2**). The amoxicillin induced-round body form was subsequently used for drug screens as described below.

### Screen for Drugs Active against Round Bodies of *B. burgdorferi*

**FIGURE 3:**
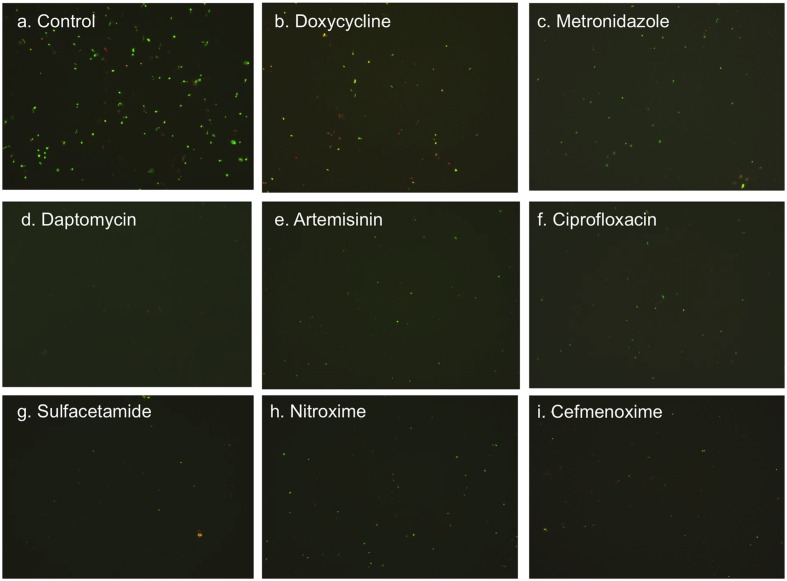
**Representative images (400 × magnification) of amoxicillin-induced round body form of *B. burgdorferi* treated with different antibiotics (labeled on the image).** A 6- day old culture was induced with 50 μg/ml amoxicillin for 72 h and subsequently treated with the listed antibiotics (50 μM) for 7 days followed by staining with SYBR Green I/PI assay and fluorescence microscopy.

Round body forms of *B. burgdorferi* are resistant to many antibiotics (Brorson et al., 2009; Sapi et al., 2011), so we used 50 μM concentration to increase the sensitivity of the FDA drug library screen. The amoxicillin-induced round bodies of *B. burgdorferi* were incubated in the 96 well plates with the library drug compounds (50 μM) for 7 days. As prior studies suggested that metronidazole and tinidazole were active against the round body form of *B. burgdorferi* (Brorson and Brorson, 1999; Sapi et al., 2011), they were included in each plate as positive controls for comparison in the drug screen. Of the 1581 FDA-library drugs tested, 23 antibiotics were found to have higher activity against the round bodies of *B. burgdorferi* than amoxicillin (46% residual viable cells) and doxycycline (42% residual viable cells) (**Table 1**). These effective hits were confirmed in further rescreens followed by fluorescence microscope counting after SYBR Green I/PI stain to verify the screening results. Among the 23 effective hits, 11 drugs (daptomycin, artemisinin, ciprofloxacin, sulfacetamide, sulfamethoxypyridazine, nifuroxime, fosfomycin, chlortetracycline, sulfathiazole, clofazimine and cefmenoxime, in order of decreasing activity) had better activity than compounds previously described with round body activity: metronidazole and tinidazole (each with 33% residual viable cells) (**Table 1**; **Figure 3**). The antimalarial drug artemisinin showed higher activity (24% residual viable cells) against the round bodies of *B. burgdorferi.* Interestingly, ciprofloxacin (28% residual viable cells) was the most active among quinolone drugs tested compared to levofloxacin 41%, norfloxacin 41%, and moxifloxacin 49%. In addition, chlortetracycline, meclocycline and rolitetracycline were more active than doxycycline (42% residual live cells) against the round bodies (**Table 1**). On the other hand, some cell wall inhibitors such as vancomycin and macrolide antibiotic carbomycin, which had reasonable activity against stationary phase *B. burgdorferi* in our previous study (Feng et al., 2014a) showed relatively weak activity against the amoxicillin-induced round bodies of *B. burgdorferi* with 38 and 43% residual live cells, respectively.

**Table 1:** Twenty-three drugs with better activity than amoxicillin against round bodies of *B. burgdorferi*^∗^.

Drugs (50 μM)	Cmax (μg/ml)^†^	Residual viable cells^‡^	Ratios of green/red fluorescence			
			
			Primary screening	Rescreening	Rescreening	*p*-Value^§^
Amoxicillin	1.5–13.8	46%	6.53	6.59	6.52	1.0000
Doxycycline	3.6–4.6	42%	6.34	6.39	6.67	0.4915
Penicillin G	1.5–2.7	38%	6.33	6.51	6.33	0.0767
Cefuroxime	2.1–13.6	34%	6.29	6.28	6.31	0.0005
Ceftriaxone	200–380	36%	6.37	6.29	6.39	0.0069
Azithromycin	2–5.2	47%	6.79	6.52	6.42	0.8116

Metronidazole	12.5–19.4	33%	6.23	6.30	6.31	0.0014

Tinidazole	40–55	33%	6.24	6.21	6.36	0.0059
**Daptomycin**^ᖮ^	**57.8–93.9**	**19%**	5.90	6.09	5.93	0.0008
**Artemisinin**	**0.3–1.6**	**24%**	5.96	6.14	6.17	0.0028
**Ciprofloxacin**	**1.2–5.4**	**28%**	6.30	6.04	6.20	0.0108
**Sulfacetamide**	**–**	**29%**	6.26	6.14	6.20	0.0011
**Sulfamethoxypyridazine**	**13.4–22.3**	**30%**	6.20	6.07	6.34	0.0149
**Nifuroxime**	**38.4–65.8**	**30%**	6.10	6.32	6.22	0.0079
**Fosfomycin**	**11.5–12.7**	**31%**	6.39	6.12	6.16	0.0191
**Chlortetracycline**	**4.33**	**31%**	6.20	6.36	6.18	0.0078
**Sulfathiazole**	**132.2 (in hen)**	**31%**	6.38	6.18	6.17	0.0141
**Clofazimine**	**0.47–0.7**	**32%**	6.29	6.22	6.24	0.0008
**Cefmenoxime**	**1.06–1.58**	**32%**	6.18	6.33	6.28	0.0050
Meclocycline		33%	6.52	6.23	6.09	0.1056
Cefmetazole		33%	6.13	6.25	6.46	0.0530
Loracarbef		33%	6.35	6.25	6.24	0.0036
Sisomicin		33%	6.27	6.12	6.45	0.0562
Sulfisoxazole		33%	6.53	6.15	6.18	0.1005
Cefazolin		34%	6.23	6.34	6.29	0.0026
Aztreonam		34%	6.16	6.38	6.33	0.0211
Thymol		34%	6.15	6.36	6.36	0.0257
Cefixime		34%	6.41	6.21	6.27	0.0169
Sulfanilate		34%	6.49	6.03	6.40	0.1649
Ceftazidime		34%	6.20	6.34	6.37	0.0126
Rolitetracycline		35%	6.23	6.35	6.37	0.0085


*^∗^Round body form of *B. burgdorferi* from 7 day old culture was treated with FDA-library drugs (50 μM) for 7 days. The dark gray shading refers to antibiotics that have been routinely recommended to treat Lyme disease. Azithromycin is a second line drug. The light gray shading includes drugs previously shown to have activity against *in vitro* round bodies (metronidazole, tinidazole) and were used as controls to identify more effective drugs. ^†^Cmax values are derived from the literature. ^‡^Residual viable *B. burgdorferi* was calculated according to the regression equation and ratios of Green/Red fluorescence obtained by SYBR Green I/PI assay. ^§^ p-values of standard t-test were calculated for the select antibiotic treated samples in comparison with the amoxicillin treated sample as a control. ^ᖮ^Bold type indicates the 11 drug candidates that had better activity against the round body forms than metronidazole or tinidazole in order of decreasing activity.*

### MIC Values of Round Body Active Antibiotics

In our previous study, we found that the activity of antibiotics against non-growing persisters was not always correlated with their activity against growing *B. burgdorferi* (Feng et al., 2014a). We therefore used log phase cultures to test the MICs of selected active hits artemisinin and ciprofloxacin that had excellent activity against the round bodies of *B. burgdorferi* using the SYBR Green I/PI assay and microscope counting. The MIC value of artemisinin was quite high at 50∼100 μg/ml for growing *B. burgdorferi*, indicating that artemisinin is much less active against growing organisms, despite its high activity against the non-growing round bodies of *B. burgdorferi* persisters. In contrast, ciprofloxacin was quite active against the growing *B. burgdorferi* with a low MIC (0.8∼1.6 μg/ml), which is in agreement with a previous study (Kraiczy et al., 2001), indicating that it is active against both growing form and non-growing round body form of *B. burgdorferi*.

### Effect of Drug Combinations on the Round Body Form and the Stationary Phase *B. burgdorferi* Persisters

**FIGURE 4:**
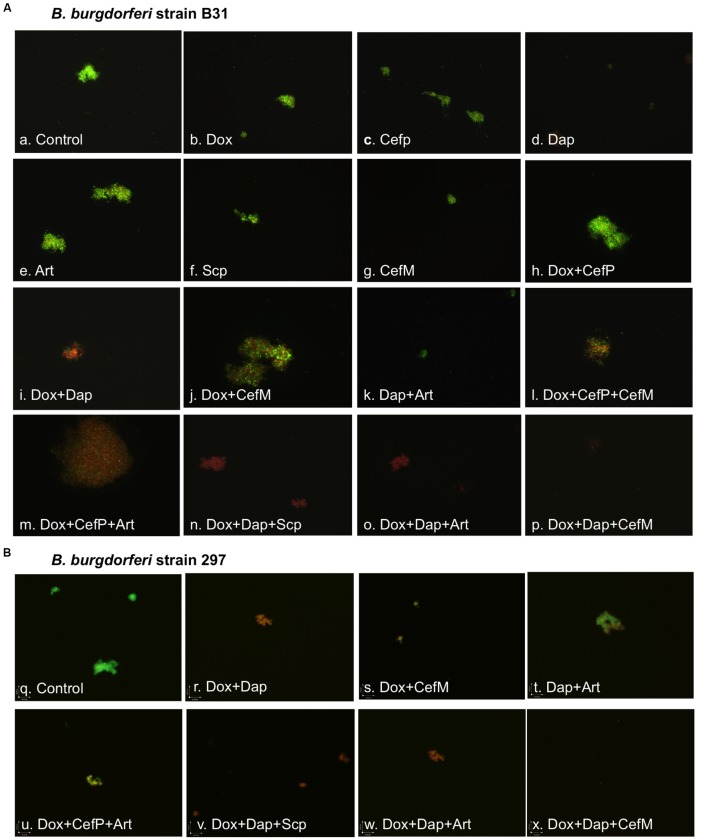
**Effect of antibiotics alone or in combinations on stationary phase *B. burgdorferi* strain B31 **(A)** and strain 297 **(B)** microcolonies.** Stationary phase culture of *B. burgdorferi* (10-day old) was treated with 10 μg/ml drugs alone or in combinations (labeled on the image) for 7 days followed by staining with SYBR Green I/PI assay and epifluorescence microscopy (200 × magnification). Green cells indicate live cells whereas red cells dead cells. Abbreviation: Dox, doxycycline; CefP, cefoperazone; Art, Artemisinin; Dap, daptomycin; CefM, cefmetazole; Scp, sulfachlorpyridazine.

We previously found that stationary phase cultures are enriched with morphological variants such as round bodies and biofilm-like aggregated microcolonies (Feng et al., 2014a, 2015). These morphological variants of *B. burgdorferi* have different antibiotic susceptibilities (Brorson et al., 2009; Sapi et al., 2011; Feng et al., 2015), and our recent study showed that some drug combinations are more effective against aggregated *B. burgdorferi* persisters than single drugs (Feng et al., 2015). To identify the best drug combinations with the active hits from the above screens against the round bodies of *B. burgdorferi*, we evaluated drugs with improved activity detected in this screening including artemisinin, cefmetazole, and sulfachlorpyridazine in combination with drugs identified in the earlier study that appeared to work well against persisters when used in combination (Feng et al., 2015). In the drug combination study, we used 10 μg/ml, a lower concentration near the achievable Cmax values of most drugs. With this approach, drug combinations were much more effective than individual drugs used alone against the round bodies (**Table 2**; **Figure 4**). Overall, the round body forms were more susceptible to the tested drugs or drug combinations than the 10 day old stationary phase culture which was enriched with more resistant aggregated microcolonies (Feng et al., 2014a, 2015) (**Table 2**). It is worth noting that antimalarial drug artemisinin highlighted itself as having among the best activity against the stationary phase *B. burgdorferi* persisters when combined with other drugs. For example, artemisinin in combination with doxycycline and cefoperazone showed excellent activity against the stationary phase *B. burgdorferi* persisters (24% residual viable cells, **Table 2**, **Figure 4**, o). Cefmenoxime and cefmetazole were the most effective of the cephalosporin drugs tested against the round body form of *B. burgdorferi*. In addition, we noted that the sulfa drug sulfachlorpyridazine when combined with daptomycin and doxycycline showed good activity against *B. burgdorferi* persisters (21% residual viable cells, **Figure 4**, n). Moreover, sulfachlorpyridazole combined with doxycycline and daptomycin showed the best activity against the round body form of *B. burgdorferi* persisters (8% residual cells, **Table 2**).

**Table 2:** Effect of drug combinations on the round body form and stationary phase culture of *B. burgdorferi* persisters^∗^

	Live cell %	CefM	Scp	Art	Nft
Control	50% (87%)	34% (53%)	38% (68%)	28% (73%)	33% (82%)
Dox	49% (72%)	31% (43%)	29% (62%)	26% (64%)	30% (74%)
CefP	30% (64%)	31% (41%)	30% (55%)	25% (42%)	25% (56%)
Dox + CefP	29% (59%)	28% (41%)^†^	25% (69%)	23% (**24%**)^‡^	23% (52%)
DAP	17% (48%)	16% (**20%**)	15% (**27%**)	24% (**29%**)	16% (33%)
DAP + Dox	16% (34%)	16% (**16%**)	8% (**21%**)	15% (**19%**)	17% (20%)


*^∗^Round body form and 10-day old stationary phase culture (values in brackets) of *B. burgdorferi* was treated with 10 μg/ml drugs or their combinations for 7 days. Residual viable *B. burgdorferi* was calculated according to the regression equation and ratio of Green/Red fluorescence obtained by SYBR Green I/PI assay as described (Feng et al., 2014a). Direct microscopy counting was employed to rectify the results of the SYBR Green I/PI assay. ^†^The best drug combinations without daptomycin are underlined. ^‡^Residual viable percentages less than 30% are shown in bold type. Abbreviation: Dox, doxycycline; CefP, cefoperazone; DAP, daptomycin; CefM, cefmetazole; Scp, sulfachlorpyridazine; Art, artemisinin; Nft, nitrofurantoin.*

We also tested some drugs and drug combinations on a different clinical isolate *B. burgdorferi* strain 297 to confirm if they also work on different strains other than strain B31. Indeed, we found no significant difference in the activity of these drugs and drug combinations for the two different strains (**Figure 4B**).

### Subculture Studies to Evaluate the Activity of Drug Combinations against Round Bodies

**FIGURE 5:**
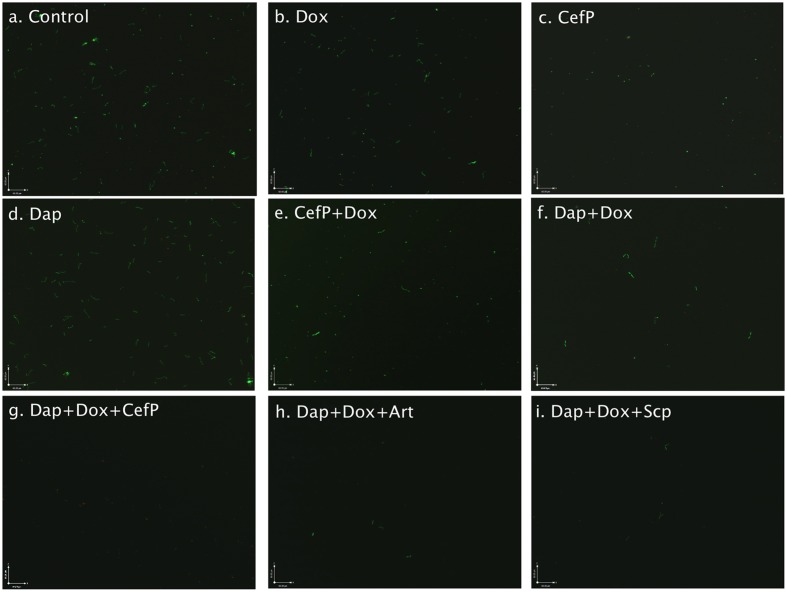
**Subculture of amoxicillin-induced round bodies of *B. burgdorferi* after treatment with different antibiotics alone or in combinations (labeled on the image).** Six day old of *B. burgdorferi* culture was induced with 50 μg/ml amoxicillin for 72 h to form round bodies, which were then treated with single antibiotics alone or in combination. Representative images (200 × magnification) were taken with fluorescence microscopy using SYBR Green I/PI staining. Only Dox + Dap + CefP completely killed all round body *B. burgdorferi* persisters as shown by lack of any viable green spirochetes after 20-day subculture. Abbreviation: Dox, doxycycline; CefP, cefoperazone; Dap, daptomycin; Art: artemisinin; Scp, sulfachlorpyridazine.

To confirm the durability of the drug combinations in killing round bodies, we performed subculture studies in BSK-H medium as described in our previous study (Feng et al., 2015). We found that drug-free round body controls and samples treated with any single drugs grew back routinely in 10-day subcultures (**Table 3**). Samples treated with two drug combinations grew more slowly (**Table 3**). However, the three drug combinations, e.g., doxycycline/daptomycin plus either cefoperozone or artemisinin or sulfachlorpyridazine did not show any sign of growth as no visible spirochetes were observed after 10-day subculture, whereas other drug combinations all had visible live spirochetes growing as observed by SYBR Green I/PI microscopy (**Table 3**). After 20-day subculture, there were about 8 × 10^6^ spirochetes in the control sample and about 5 × 10^6^ spirochetes in doxycycline-treated samples (**Table 3**). Daptomycin alone, or two drug combinations doxycycline/cefoperazone and doxycycline/daptomycin could not sterilize the round bodies of *burgdorferi*, as they all had visible spirochetes growing after 20 day subculture (**Figure 5**). However, doxycycline/daptomycin and either artemisinin or sulfachlorpyridazine significantly reduced the number of spirochetes with very few spirochetes being visible after 20 day subculture (**Figure 5**). As in our previous study (Feng et al., 2015), daptomycin in combination with doxycycline and cefoperazone also showed the best activity which killed all round body form of *B. burgdorferi* persisters with no viable spirochetes observed after 20 day subculture (**Figure 5**). In a separate experiment, we found BSK-H medium stored under experimental conditions for 20 days showed no loss or inactivation of nutrients, since *B. burgdorferi* displayed the same normal exponential growth in either 20 day old medium or fresh medium.

**Table 3:** Subculture following exposure to single drugs or drug combinations to assess the viability of remaining round body forms of *B. burgdorferi.*^∗^

Drugs^†^	Residual viable cells^‡^	Spirochetes number after 10 days subculture^§^	Spirochetes number after 20 days subculture^§^
Control	49%	2 × 10^6^	8 × 10^6^
Dox	44%	1 × 10^6^	5 × 10^6^
Dox + Dap + CefP	13%	<1 × 10^5^	<1 × 10^5^^ᖮ^
Dox + Dap + Art	15%	<1 × 10^5^	3 × 10^5^
Dox + Dap + Scp	17%	<1 × 10^5^	6 × 10^5^
Dox + CefP	31%	7.5 × 10^5^	4 × 10^6^
Dap	18%	5 × 10^5^	5 × 10^6^
CefP	33%	9 × 10^5^	4 × 10^6^


*^∗^Amoxicillin induced round body form *B. burgdorferi* culture (500 μl) was treated with 10 μg/ml drugs alone or drug combinations for 7 days. Then, 50 μl of washed bacterial cells was subcultured in 1 ml fresh BSK-H medium for 10 and 20 days, respectively and examined by microscopy. ^†^Abbreviations: Dox, doxycycline; CefP, cefoperazone; Dap, daptomycin; Art, artemisinin; Scp, sulfachlorpyridazine. ^‡^Green/Red fluorescence ratios were obtained by microplate reader after SYBR Green I/PI staining. Each value is the mean of three replicates. ^§^The number of spirochetes was evaluated by microscope count. ^ᖮ^Below the detection limit as shown by lack of any visible spirochetes by microscopy.*

## Discussion

Previous *in vitro* studies showed that the round body form of *B. burgdorferi* as a persister form could survive in adverse conditions including antibiotic exposure in culture (Kersten et al., 1995; Brorson et al., 2009; Barthold et al., 2010). Although metronidazole, tinidazole and tigecycline were reported individually to have activity against these round body forms, they were unable to completely eradicate these persisters in culture (Sapi et al., 2011). In this study, to identify more effective drugs active against round bodies, we first established an amoxicillin-induced round body model of *B. burgdorferi* persisters and then screened an FDA-approved drug library for highest activities against the round bodies. We were able to identify 23 drug candidates that are more active than doxycycline or amoxicillin, 11 of which had better activity against the round body form of *B. burgdorferi* (**Table 1**) than metronidazole or tinidazole, which were previously cited as having such abilities to kill round body forms (Brorson and Brorson, 1999; Sapi et al., 2011).

We used a relatively high concentration (50 μM) of drug candidates in the drug screen. A major reason to do so is that lower concentration of drugs (10 μM commonly used in different drug screens) in our previous studies identified very few drug candidates because stationary phase cultures or round body forms induced by amoxicillin are quite resistant to drugs or antibiotics. This necessitates the use of higher drug concentrations to identify any drug candidates with activity against these resistant forms. Some of the identified drug candidates though show weak activity on their own, can be quite active in the drug combinations (Feng et al., 2015, 2016). Thus, an initial relatively high drug concentration would be helpful to identify these drugs in the high throughput screen, and this is followed by use of a lower concentration (10 μg/ml) for each candidate drug in drug combination tests which show good activity. It is worth noting that most drugs’ Cmax values are higher than or close to this 10 μg/ml concentration used in this study.

In a previous study, we identified several drugs that show excellent activity against stationary phase *B. burgdorferi* from an FDA drug library (Feng et al., 2014a). Replication of activity against *B. burgdorferi* round body persisters by daptomycin, clofazimine and sulfa drugs in this study validates our previous findings that such drugs are active against persisters in the stationary phase model (Feng et al., 2014a). Importantly, we also identified additional drugs active against the amoxicillin-induced round bodies of *B. burgdorferi* that did not show good activity in the previous drug screen against the stationary phase *B. burgdorferi* (Feng et al., 2014a). These include artemisinin, ciprofloxacin, nifuroxime, fosfomycin, tinidazole, loracarbef, and thymol that appear to have enhanced activity against the round body form. The reason why these new drug candidates are identified that are distinct from the previous screen on stationary phase culture without antibiotic treatment is presumably a reflection of the difference in the amoxicillin-induced round bodies being used in this screening process. Nevertheless, it is interesting that despite the difference of the two models, some common drug candidates such as daptomycin, clofazimine and sulfa drugs were found to be active in both persister models. These findings suggest the two persister models, i.e., stationary phase culture and amoxicillin-treated, may have overlapping persister cell subpopulation characteristics among the very heterogeneous persister population (Feng et al., 2014a; Zhang, 2014). Both may be useful methods for persister drug screenings.

As in our previous study (Feng et al., 2014a), daptomycin remains the most active drug against the round bodies of *B. burgdorferi.* Daptomycin killed most planktonic round body form of *B. burgdorferi* (**Figure 3b**). It is possible that daptomycin preferentially acts on the membrane of the round body form of *B. burgdorferi* that is different from the membrane of actively growing spirochetal form. Daptomycin is known to disrupt the *Staphylococcus aureus* membrane structure and cause rapid depolarization thus depleting membrane energy that may be required for viability of the persisters (Pogliano et al., 2012). The drug has been shown to work more effectively in combination with beta-lactams, for example, against antibiotic resistant enterococci (Brown et al., 2015).

An interesting finding of the study is the observation that the antimalarial drug artemisinin also showed excellent activity against the round body form of *B. burgdorferi* persisters (**Figure 3e**). It is worth noting that artemisinin similar to daptomycin has a high MIC (50–100 μg/ml) against growing *B. burgdorferi*, while showing excellent activity against the round bodies of *B. burgdorferi.* Artemisinin is a commonly used antimalarial drug isolated from the wormwood plant *Artemisia annua*, a Chinese herbal medicine. The mechanism of artemisinin action is not well understood. The antimalarial activity of artemisinin might involve endoperoxide activation by free ferrous iron from hemoglobin digestion by malaria parasites (Wells et al., 2009). However, the content of ferrous iron or hemoglobin is very low in the *B. burgdorferi* culture (Rodriguez et al., 2007), so the activation of endoperoxide might not be the main mechanism of artemisinin activity against *B. burgdorferi* round bodies. In yeast, artemisinin impairs the membrane structure and causes depolarization of the mitochondrial membrane (Li et al., 2005; Wang et al., 2010). In this respect, it is possible that artemisinin may have a similar mechanism of action as daptomycin in disrupting the bacterial membrane as the basis for its high activity against the round bodies of *B. burgdorferi*.

We previously found daptomycin combined with doxycycline and cefoperazone could best eliminate the most resistant biofilm-like microcolonies of *B. burgdorferi* (Feng et al., 2015, 2016). In this study, we found artemisinin was the best alternative drug in combination with doxycycline and cefoperazone compared to daptomycin-containing regimen and showed excellent activity against the amoxicillin-induced round body form of persisters (**Table 2**; **Figure 3c**). We also noticed the lipophilic antibiotic clofazimine, which has complex antimicrobial activity including membrane disruption and depolarization (Van Rensburg et al., 1992; Cholo et al., 2012), also showed good activity against both round body form and stationary phase persisters (Feng et al., 2014a). The results using artemisinin, clofazimine and daptomycin (Feng et al., 2014a) suggest membrane disruption may be a good approach to killing *B. burgdorferi* persisters.

Besides the top-ranked hits of screened drugs, we found many sulfonamide antibiotics such as sulfacetamide, sulfamethoxypyridazine and sulfaquinoxaline are highly active against the round body form (**Table 1**). The sulfonamide antibiotics have also been identified in the previous drug screen against stationary phase persisters and showed low MICs (<0.2 μg/ml) (Feng et al., 2014a). The sulfonamides inhibit utilization of PABA required for the synthesis of folic acid, which results in blockade of several enzymes needed for synthesis of DNA and methionine, glycine, and formylmethionyl-transfer-RNA. Because methionine is a methyl group donor, sulfa drugs may also inhibit methylation process in *B. burgdorferi*, which may be important for their anti-persister activity. It is worth noting that sulfachloropyridazine as an analog of sulfamethoxypyridazine also showed good activity against stationary phase *B. burgdorferi* (residual viable cells about 38%). When combined with daptomycin/doxycycline this trio showed remarkable activity against stationary phase *B. burgdorferi* (residual viable cells is about 8%) (**Table 2**). We believe that further studies on metabolic changes of the round body form of *B. burgdorferi* could help understand the mechanisms by which sulfa drugs act against *B. burgdorferi* persisters.

In addition to the previously described daptomycin, clofazimine, cefoperazone and, sulfa drugs (Feng et al., 2014a), we also discovered some additional drugs preferentially active against the amoxicillin-induced round bodies of *B. burgdorferi*. The fluoroquinolone drug ciprofloxacin has previously been shown to be active against *B. burgdorferi in vitro* and could kill the inoculum at a relatively high concentration of 16 μg/ml minimum bactericidal concentration (MBC) (41.5 μM) after 72 h incubation (Kraiczy et al., 2001). Here we found that ciprofloxacin was the most active of the fluoroquinolones against the round bodies of *B. burgdorferi*, but it was not identified to have activity against *B. burgdorferi* stationary phase persisters in our previous screen (Feng et al., 2014a) as it was not in the prior version of the FDA drug library (version 1.1). However, ciprofloxacin (50 μM) alone could not completely kill the round bodies after 7 days. This indicates that the round body form is more resistant or tolerant to this antibiotic than multiplying *B. burgdorferi.* On the other hand, we also noticed some drugs such as nifuroxime and thymol did not show activity in the previous drug screens on stationary phase *B. burgdorferi* (Feng et al., 2014a, 2015), but showed good activity against the round body form in this study. This specific activity against the round body form could be related to the physiological difference of different morphological forms and/or the synergistic activity of these drugs with amoxicillin used to induce round forms used for drug screens. It is of interest that chlortetracycline was more active than doxycycline against the round body forms and that nitrofuran derivative nifuroxime was more active than metronidazole or tinidazole (**Table 1**). These findings could indicate the side chain involved in both cases may have conferred additional activity against the round body persisters.

We did the drug combination test on the stationary phase *B. burgdorferi* using the nifuroxime analog nitrofurantoin (residual live cell, 39%), and the results showed that nitrofurantoin combined with cefoperazone was more effective than each drug alone (**Table 2**). Likewise, the natural antimicrobial thymol combined with amoxicillin showed good activity (residual percentage, 34%) in the round body drug screen but thymol alone did not work on the stationary phase *B. burgdorferi* (residual live cell percentage, 82%) in the previous drug screen. Palaniappan and Holley (2010) reported that thymol could reduce the resistance in *E. coli* and *S. aureus* to ampicillin and penicillin. This synergistic activity between thymol and beta-lactams may explain its activity against the *B. burgdorferi* round body form. These results suggest that the drug combination could be an effective approach if *B. burgdorferi* persisters are playing a significant role.

However, some drugs that had activity against stationary phase *B. burgdorferi* such as beta-lactams, vancomycin, streptomycin, and amphotericin B (Feng et al., 2014a) did not show good activity against the round body form (**Table 1**). However, we noted two cephalosporins, cefmenoxime and cefmetazole showed good activity against the round body form of *B. burgdorferi*. In the previous drug screen on stationary phase *B. burgdorferi*, cefoperazone, which was the best cephalosporin for killing stationary phase *B. burgdorferi*, also had certain activity against the round body form (not shown). Future studies are needed to explore the mechanism of action of these cephalosporins that have activity against the round body *B. burgdorferi* persisters which may involve targets beyond the cell wall synthesis. Vancomycin is a glycopeptide antibiotic acting on the cell wall rather than acting on the cell membrane like daptomycin. We did not find good activity of vancomycin against amoxicillin treated round bodies, though it showed relatively good activity against stationary phase *B. burgdorferi* in the previous drug screen (Feng et al., 2014a). This might be due to the relative cell wall deficiency state of the round body form induced by amoxicillin.

Despite the description of drug candidate’s active against the round body forms, their significance in improving the treatment remains to be determined. While evidence that round body form of persisters examined in this study may occur *in vivo* during infection remains to be confirmed, it is also unknown if antibiotics that target such potential persisters would lead to improved outcomes in human infection compared with standard Lyme disease treatment with doxycycline or amoxicillin that do not have good activity against the round body forms of *B. burgdorferi* (Brorson et al., 2009; Barthold et al., 2010) or if they even induce such forms *in vivo.* These studies indicate that it would be difficult to kill the round body form of *B. burgdorferi* using the current Lyme antibiotics if similar organisms exist *in vivo* or are incompletely eradicated in the human host under environmental stressors. The drug candidates identified in this study could allow the above possibility to be tested in animal models of persistence using the drug combinations that are active against round body forms of *B. burgdorferi* in the future.

In summary, this study represents the first high throughput drug combination screen using an FDA library against amoxicillin-induced round body forms of *B. burgdorferi*. Several FDA-approved drugs show excellent activity against such forms. Drug candidates that are preferentially active against the round bodies include artemisinin, ciprofloxacin, nifuroxime, fosfomycin, chlortetracycline, and some sulfa drugs. We found that triple drug combinations artemisinin/cefoperazone/doxycycline, daptomycin/sulfachlorpyridazine//doxycycline and daptomycin/cefoperazone/ doxycycline had among the best activity against both the round body model and the stationary phase persister model. These round body effective drugs and drug combinations provide new candidates for further evaluation in animal studies and potentially clinical investigation to determine whether drug combinations could improve resolution of symptoms in those patients who do not appropriately or completely respond to traditional antibiotic therapy for Lyme disease.

## Conclusion

In this study, we performed a drug combination screen using an FDA drug library on amoxicillin-induced round bodies of *B. burgdorferi* that identified 23 drug candidates with higher activity than either amoxicillin or doxycycline. Eleven of the 23 candidates had higher anti-round body activity than either metronidazole or tinidazole. Although some drug candidates such as daptomycin and clofazimine overlapped with a previous screen against stationary phase *B. burgdorferi*, new active drug candidates identified include artemisinin, ciprofloxacin, nifuroxime, fosfomycin, chlortetracycline, sulfacetamide, sulfamethoxypyridazine and sulfathiozole that have high activity against round body forms. We found two triple drug combinations artemisinin/cefoperazone/doxycycline and sulfachlorpyridazine/daptomycin/doxycycline, which had the highest activity against round bodies and stationary phase *B. burgdorferi* persisters. Further *in vivo* animal and human studies are needed to evaluate the significance of these findings for improved treatment of Lyme disease.

## Author Contributions

YZ conceived the experiments; JF, WS, SZ, and DS, performed the experiments; JF, DS, and YZ analyzed the data; and JF, PA, and YZ wrote the paper.

## Conflictof Interest Statement

The authors declare that the research was conducted in the absence of any commercial or financial relationships that could be construed as a potential conflict of interest.
